# Estimating the efficacy and plume reach of semiochemical‐baited traps on the capture of *Trogoderma granarium* everts

**DOI:** 10.1002/ps.8805

**Published:** 2025-04-08

**Authors:** Paraskevi Agrafioti, William R. Morrison, Michael Domingue, Evagelia Lampiri, Scott W. Myers, Christos G. Athanassiou

**Affiliations:** ^1^ Laboratory of Entomology and Agricultural Zoology, Department of Agriculture, Crop Production and Rural Environment University of Thessaly Nea Ionia Greece; ^2^ United States Department of Agriculture, Agricultural Research Service, Center for Grain and Animal Health Research Manhattan Kansas USA; ^3^ United States Department of Agriculture—Animal and Plant Health Inspection Services—Plant Protection and Quarantine, Science and Technology Buzzards Bay Massachusetts USA

**Keywords:** khapra beetle, *Trogoderma granarium*, traps, attractants, captures, monitoring

## Abstract

**BACKGROUND:**

The khapra beetle, *Trogoderma granarium*, is a serious pest of stored grains that infests more than 100 different commodities. The current wall trap used for monitoring is optimized for the behavior of khapra beetles. In this study, we tested existing and modified trap designs for *Trogoderma* after release of *T. granarium* exclusively. The work aimed to evaluate trap efficacy, while providing a template for further attract‐and‐kill research. Three bioassays were conducted in nine rooms. Four traps were evaluated: dome, wall, modified wall, and the Wilkins interception trap.

**RESULTS:**

Based on our results, dome traps collected significantly more individuals compared to the other traps, whereas the modified wall and Wilkins traps collected significantly fewer individuals compared to the dome trap. Regarding progeny production, the highest number of individuals was found from a release distance of 0.1 m. The highest number of individuals was captured at 0.1 m.

**CONCLUSIONS:**

The most effective trap was the dome trap. The plume reach and distance from the trap/attractant source is an important parameter that can affect trapping efficacy in monitoring programs. © 2025 The Author(s). *Pest Management Science* published by John Wiley & Sons Ltd on behalf of Society of Chemical Industry. This article has been contributed to by U.S. Government employees and their work is in the public domain in the USA.

## Graphical abstract

"Simulated warehouse": In this study, we take the step of testing existing and modified trap designs for *Trogoderma* after the release of *T. granarium* exclusively. This work will serve to evaluate trap efficacy, while providing a template for further attractant–kill research. The most effective trap was dome trap. The distance from the trap/attractant source is an important parameter that can affect trapping efficacy.

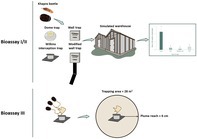

## 1 INTRODUCTION

The khapra beetle, *Trogoderma granarium* Everts (Coleoptera: Dermestidae), is a major quarantine insect species of stored products that infests more than 100 different commodities and substrates, ranging from grains to milk powder.^1^, ^2^, ^3^, ^4^, ^5^, ^6^ Apart from its wide food preferences, this species can develop at temperatures that exceed 35 °C, which is considered unsuitable for other major stored product insect species with which they might directly compete.^7^, ^8^ Nevertheless, even when *T. granarium* is simultaneously present with other stored product insect species on the same commodity, it can easily dominate, provided that the prevailing conditions, especially temperature, are suitable.^8^ Although they can develop rapidly at high temperatures, larvae of *T. granarium* can survive for a long period that may exceed several years, evewhen temperatures are low (below 30 °C), which also contributes to their cryptic distribution in sensitive locations.^2^, ^9^, ^10^, ^11^, ^12^, ^13^, ^14^

Considering the importance of grains and other durable commodities for global food security and the phytosanitary regulations for international trade, early detection of incipient risks is essential. In this context, different traps have been developed for monitoring of stored product insects, such as probe traps that are mostly used for the detection of beetles inside bulked grains.^15^, ^16^, ^17^ There are aerial pheromone‐baited traps for moths^18^, ^19^ or for flying beetles, such as *Lasioderma serricorne* (F.) (Coleoptera: Ptinidae).^20^, ^21^ Apart from early detection, these traps have proved valuable in estimating the population densities of different stored product insect species and for applying time control measures, based on their spatio‐temporal distribution.^22^ Moreover, many traps will simultaneously capture many stored product insect species that coexist and co‐infest the product.^23^, ^24^

The flightless nature of *Trogoderma granarium* provides additional crypsis for interception efforts.^4^, ^8^, ^10^, ^25^ As such, this species cannot reach aerial traps, but only traps that can be crawled into from the floor (e.g., dome traps, Storgard; Trécé Inc., Adair, OK, USA) or wall^26^ surfaces. In addition, adults of this species have been been found to utilize a sex pheromone, which is a mixture of 92% *Z* and 8% *E* isomers of 14‐methyl‐8‐hexadecenal and is also an effective attractant for many other species of the genus *Trogoderma*.^27^ It was also determined that late‐stage *T. granarium* larvae are attracted to commercially available pheromone lures (KB/WHB, Storgard Cap; Trécé, Inc.).^28^ The multispecies detection capacity of the lure can be problematic for *T. granarium* management programs because the targeted *Trogoderma* species include several that are difficult to distinguish by morphological characteristics. Moreover, the larvae of *Trogoderma* spp., which are more common in floor traps, are even more difficult to identify. Because *T. granarium* spends most of its lifespan at the larval stage, the detection of larvae in traps is more likely than short‐lived adults.^29^, ^30^

Considerable research been conducted to evaluate different traps and attractants for the detection of this species.^28^, ^31^, ^32^, ^33^ Based on the data that have been obtained so far, even at close range, *T. granarium* does not enter floor traps with a high degree of frequency. In a recent study, Sakka *et al*.^33^ tested both dome and box (Insects Limited, Inc., Westfield, IN, USA) floor traps in laboratory pheromone‐baited experiments with adults and larvae of this species. In that work, the authors reported that this poor trap performance was exhibited regardless of the trap type and the presence or absence of a pheromone lure.^33^ Similarly, Gourgouta *et al*.,^32^ in a series of laboratory trials with dome traps, found that both *T. granarium* and *T. variabile* did not respond well. Thus, all the data available so far suggest that trapping of *T. granarium* is problematic, with low detection rates being likely in intensively monitored areas where pests are active. Highlighting this challenge are studies such as Castane *et al*.,^31^ which successfully used five types of floor traps in Spain for monitoring *Trogoderma* spp., but detected no *T. granarium* individuals. When the capture rates are low in traps, it is difficult to rule out that a particular species may be present.

Furthermore, the United States Department of Agriculture uses wall traps in its program monitoring activities, which are baited with a pheromone lure along with wheat germ, for larval detection. These wall traps have been utilized in an extensive surveillance effort in the USA.^28^, ^34^, ^35^ They rely on *T. granarium* adults and larvae to climb up wall surfaces to reach the trap, which presumably will make it more difficult for crawling adults and larvae to reach these traps. Indeed, recent research has suggested *T. granarium* has poor climbing ability, especially when surfaces approach perpendicular to the plane of walking.^35^ This may hinder the efficacy of the standard wall trap, which is commonly used for surveillance efforts of *T. granarium* and placed on walls 4 cm up. A comparison of the efficacy of these wall traps *versus* floor trap designs for *T. granarium* has never been performed in field conditions. Quantifying the relative efficacy of the different trap designs in realistic conditions has never been accomplished.

This overview of the recent methods for trapping to detect and estimate *T. granarium* presence in storage demonstrates the general challenge of the task. The occurrence of *T. granarium* should be predefined in the areas where these modalities are to be tested. Restricted semifield tests in partially controlled environments can be a solution to this problem, with release of known insect numbers that can be potentially recovered in the traps. Establishing the parameters for semifield trapping can lead to further modifications of trap design, and the identification of killing agents and attractants. A range of goals might be achieved by experimentation in such semifield conditions beyond improving detection capability to ultimately encompass attract‐and‐kill strategies. Earlier studies have illustrated that attract‐and‐kill can be a promising control method in storage and processing facilities for the control of different species.^24^ In this study, we tested existing and modified trap designs for *Trogoderma* after release of *T. granarium* exclusively. The work was intended to evaluate trap efficacy, while providing a template for further attract‐and‐kill research.

## 2 MATERIALS AND METHODS

### 2.1 Insects

The population of *T. granarium* used in the experiments was reared at the Laboratory of Entomology and Agricultural Zoology (LEAZ), Department of Agriculture, Crop Production and Rural Environment, University of Thessaly, Greece, on whole wheat kernels in incubators (210 × 168 × 90 cm) set at 25 °C, 65% relative humidity, and continuous darkness. Adults and larvae of mixed age were used in all bioassays.

### 2.2 Bioassay I

**Figure 1:**
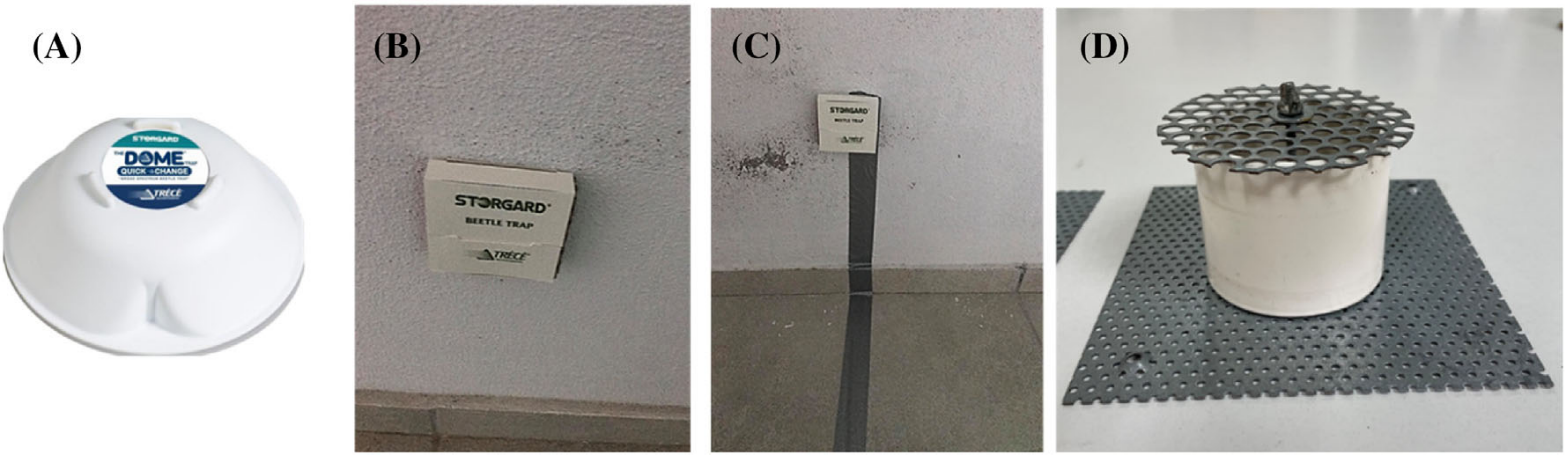
The trap types used in the bioassays: (A) dome trap, (B) wall trap (unmodified wall trap), (C) modified wall trap, and (D) Wilkins trap.

Three different types of traps were assessed for this bioassay: a dome trap (Trécé, Inc.), an unmodified wall trap (Trécé, Inc., Oklahoma, USA), and a modified wall trap (Fig. 1(A)–(C)). For the first bioassay, Gorilla tape (grey, 18 × 10 × 14 cm, Gorilla Glue, USA) was used to provide a tactile and visual guide starting 10 cm perpendicular to the wall, and leading 10 cm up the wall from the floor and under the placement of the modified wall trap (Fig. 1(C)). All traps contained a rubber septum pheromone lure (Trécé, Inc.) and wheat germ (a teaspoon) as a kairomone attractant (Trécé, Inc.). The traps were placed in four rooms with dimensions of 5.85 × 3.90 × 3 m. Each room contained six traps, including two replicates of each of the three trap types. Within each room, in two opposite corners, dome traps were placed 10 cm away from the walls. The wall traps were placed near the other two corners. A pair of unmodified wall traps and a pair of modified wall traps were placed 1 m from these corners and 1 m above the floor. A total of *n* = 7 releases of unsexed adults and larvae of *T. granarium* were performed by placing an infested diet into each room. On a weekly basis 40 g of the diet (e.g., with 100 unsexed individuals consisting of adults and larvae) was placed in a small colander with a coffee filter inside to provide a favorable location for the insects to wait until they decided to disperse and to ease clean up later. In each release, a consistent volume of infested material was used. Every 7 days, the wheat kairomone of each trap was collected and transferred to a freezer at LEAZ to count the number of captured individuals. New wheat kairomone was placed in each trap on each collection. The pheromone lures in each trap were replaced after 1 month.

### 2.3 Bioassay II

The traps utilized in this bioassay were a Wilkins interception trap (WIT) (Fig. 1(D)), as described by Wilkins *et al*.,^23^ an unmodified wall trap (Trécé, Inc.), a modified wall trap, and a dome trap (Trécé, Inc.). In the modified wall trap, we used packing tape (yellow, 50 × 66 mm; 3M Scotch, USA) with a paper surface (purchased in a hardware store in Greece). The lures were the same as described for bioassay I in rooms 5 and 6 (with the same dimensions as described above). The four types of traps were placed along the perimeter of the room (each corner), spaced evenly apart, and randomly assigned with wall traps were placed 1 m above the floor. In this case, a different release protocol was followed that allowed a more precise calculation of the recapture rate and independence of replicates. A total of *n* = 8 releases of 50 unsexed adults and 50 larvae of *T. granarium* per release were performed, with new releases every 7 days. At the end of each 7‐day period, the experimental rooms were swept of remaining non‐responders, which were removed to avoid affecting subsequent replicates. Traps were checked once a week by removing their receptacles (kairomone and pheromone) and transferred to LEAZ, where the total numbers from each life stage were counted.

### 2.4 Bioassay III

Three other semifield rooms, with the same dimensions as described above, were used (e.g., rooms 7, 8, and 9). One room was assigned to a single WIT trap with pheromone and wheat kairomone placed in the center of the room, the second room contained a WIT trap with wheat kairomone only, and the third contained a WIT trap without any pheromone or wheat kairomone lures. Fifty adults and 50 larvae of *T. granarium* were placed in each cardinal position relative to the trap at fixed distances. The distances included individuals deployed at 0.1, 0.5, 1.0, and 1.9 m from the WIT trap. One distance was deployed at a time and on a given deployment, all cardinal points for a distance were deployed simultaneously. A total of *n* = 3 releases of 50 adults and 50 larvae of *T. granarium* were performed for each distance, with new releases every 3 days. At end of each 3‐day period, the experimental rooms were swept of remaining non‐responders, which were removed to avoid affecting the next replicates. Traps were checked every 3 days by retrieving the trap, placing it with its container in a sealable zip lock, and transferring it to LEAZ, where the total numbers of adults, pupae, and larvae were recorded. After each measurement, all living individuals were placed in cylindrical plastic vials (3 cm in diameter, 8 cm high; Rotilabo Sample tins Snap on lid, Carl Roth, Germany) with 20 g of soft wheat for 6 weeks so that the progeny production could be counted to assess whether the individuals captured in the WIT trap were capable of oviposition at that particular trap.

### 2.5 Statistical analysis

In all bioassays, data were first checked for normality and homogeneity using Shapiro–Wilk's and Levene's tests, respectively.^36^ Since the numbers of the different life stages that were captured, as well as the low rates of captured individuals, were non‐normally distributed, non‐parametric analysis was used. The data were compared with a Kruskal–Wallis *H*‐test followed by a multiple pairwise Mann–Whitney *U*‐test. Significant values were adjusted by the Bonferroni correction for multiple tests.^36^ For each bioassay room, date, trap type, and check date effects on captured individuals were analyzed using a generalized linear model, assuming a poison distribution and logit function, in the Statistical Package for the Social Sciences (SPSS) Statistical Package (SPSS v.26, IBM). In bioassay I, the Kruskal–Wallis test was used to determine the differences among the trap types in each room. In bioassay II, the Kruskal–Wallis test was used to determine the differences among the trap types. In bioassay III, the Kruskal–Wallis test was used to define the differences among the distances (0.1, 0.5, 1.0, and 1.9 m) in each room. In addition, for bioassay III, the Miller–Adams–McGhee (MAG) slope method defined in Miller *et al*.^41^ was used to calculate the plume reach of the Wilkins interception traps. Plume reach was defined as the distance from a baited trap that elicits a behavioral response from an insect randomly foraging through the environment.^41^ In particular, the adults and larvae were pooled among releases within treatments (e.g., rooms) and plotted as follows: (i) untransformed proportion of *T. granarium* recaptured at a specific distance (mean spT_fer_); (ii) inverse proportion of *T. granarium* recaptured at a specific distance (1/mean spT_fer_; MAG plot); and (iii) spT_fer_ × annulus area of release *versus* the release distance from the trap (Miller plot). In addition, the trapping radius (plume reach + maximum dispersive capacity of animal in time period) and trapping area (area in which all the organisms with a measurable probability of being attracting and reaching a trap) were calculated from Miller plots. SPSS version 26 software (IBM) was used to perform the statistical analyses.

## 3 RESULTS

### 3.1 Bioassay I

**Figure 2:**
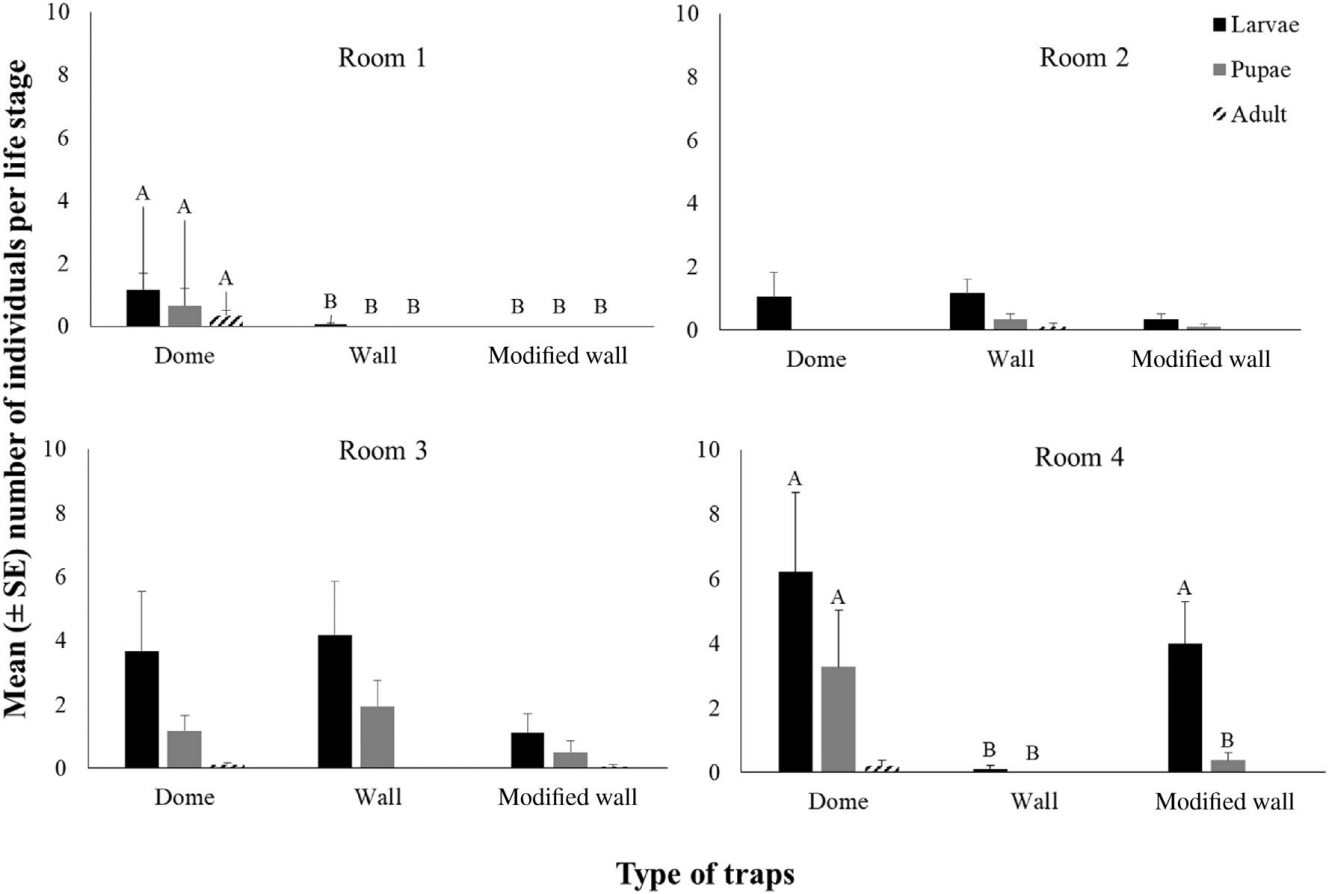
The mean (± standard error [SE]) number of individuals per life stage (larvae, pupae, and adult), depending on the trap type (dome, wall, and modified wall) for each room (1, 2, 3, and 4). Within each room and life stage, means followed by the same letter do not differ significantly, according to Kruskal–Wallis test, at *P <* 0.05. Where there are no letters, no significant differences were noted. Statistical parameters for room 1 were at larvae test statistic 10.292, *P* = 0.006, at pupae test statistic 6.231, *P* = 0.044, and at adult test statistic 8.467, *P* = 0.014 and for room 4, at larvae were test statistic 13.266, *P* = 0.001 and at pupae were test statistic 11.369, *P* = 0.003. In all cases, df = 2.

**Figure 3:**
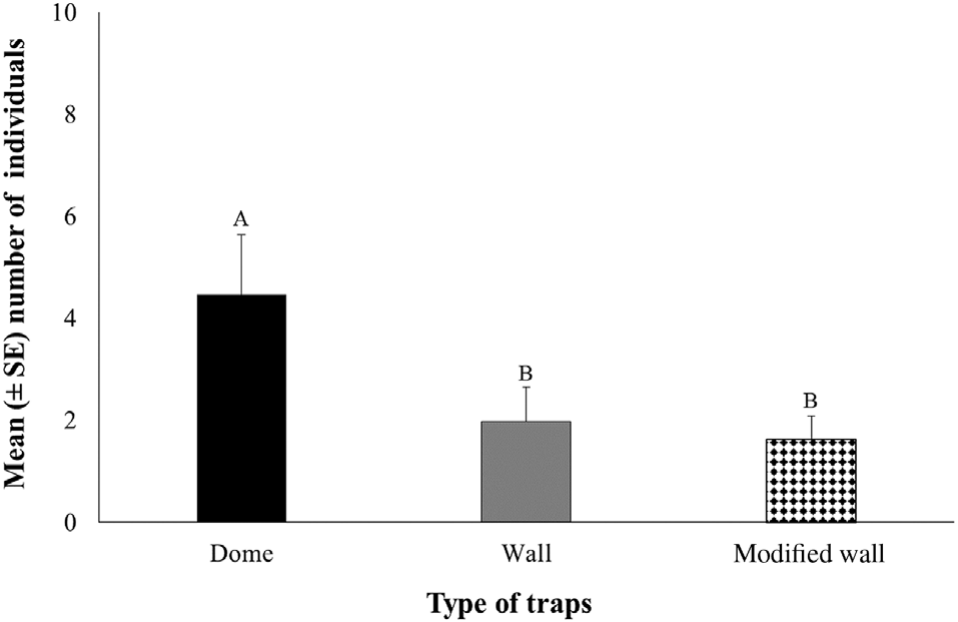
The mean (± standard error [SE]) number of all life stages collected in dome, wall, and modified wall traps. Means followed by the same letter do not differ significantly, according to the Kruskal–Wallis test, at *P* < 0.05. Statistical parameters were recorded with test statistics 8.110, *P* = 0.017.

**Figure 4:**
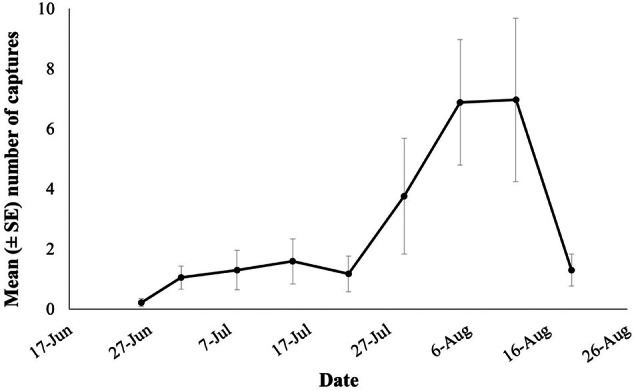
The mean (± standard error [SE]) number of captures for all life stages of *Trogoderma granarium* over time for 2020, experimental period from June to August.

The mean number of individuals per life stage (larvae, pupae, and adult) according to trap type (dome, wall, and modified wall) is presented in Fig. 2. The main effects (of trap, room, and check date) as well as their two‐way and three‐way interactions (trap × room, trap × check date, and room × check date) affected the number of individuals caught in the traps (Table 1). In general, most individuals were collected at the larval stage (Fig. 2). Significant differences were recorded among the trap types in rooms 1 and 4 (Fig. 2). Specifically, in room 1, there were zero captures in the modified wall trap, while in room 4, more than three individuals were captured (Fig. 2). Most of the larvae were caught in the dome trap (six individuals) (Fig. 2). However, the insect captures by the dome traps were not significantly affected by room (Table 1 and Fig. 2) in contrast to the insect captures by the unmodified and modified wall traps, which appeared to be influenced by room (Table 1 and Fig. 2). Dome traps collected significantly more individuals compared to the unmodified and modified wall traps (Fig. 3). In total, 350 individuals were captured in traps for the 2020 experimental period, with the highest number of captures recorded during August (Fig. 4).

**Table 1:** Generalized linear model showing the main effects and their interactions of captures of *Trogoderma granarium* in traps for bioassay I.

Main effects and interactions	df	Wald *x* ^2^	*P*
Whole model	107	1448.14	<0.001
Intercept	1	21.15	<0.001
Trap	2	11.00	0.004
Room	3	23.63	<0.001
Check date	8	70.57	<0.001
Trap × room	5	18.17	0.003
Trap × check date	12	44.95	<0.001
Room × check date	15	63.86	<0.001
Trap × room × check date	8	10.31	0.244

### 3.2 Bioassay IΙ

**Figure 5:**
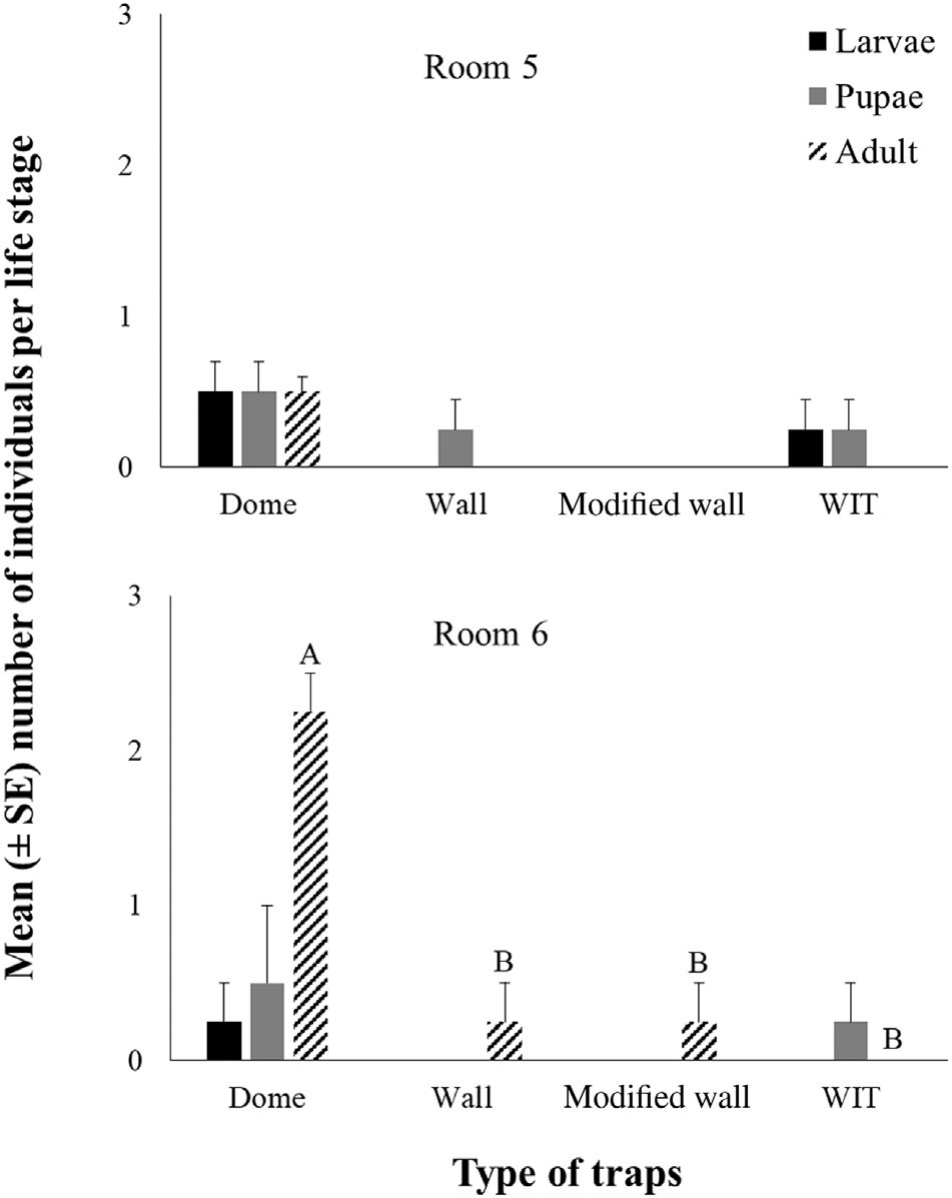
The mean (± standard error [SE]) number of larvae, pupae, and adults collected in dome, wall, modified wall, and Wilkins interaction trap (WIT) traps for each room. Within room and life stage, means followed by the same letter do not differ significantly, according to the Kruskal–Wallis test, at *P <* 0.05. Where there are no letters, no significant differences were noted. Statistical parameters were noted for room 6 at adult test statistic 11.647, *P* = 0.009. For each room, df = 3 and total *N* = 26.

**Figure 6:**
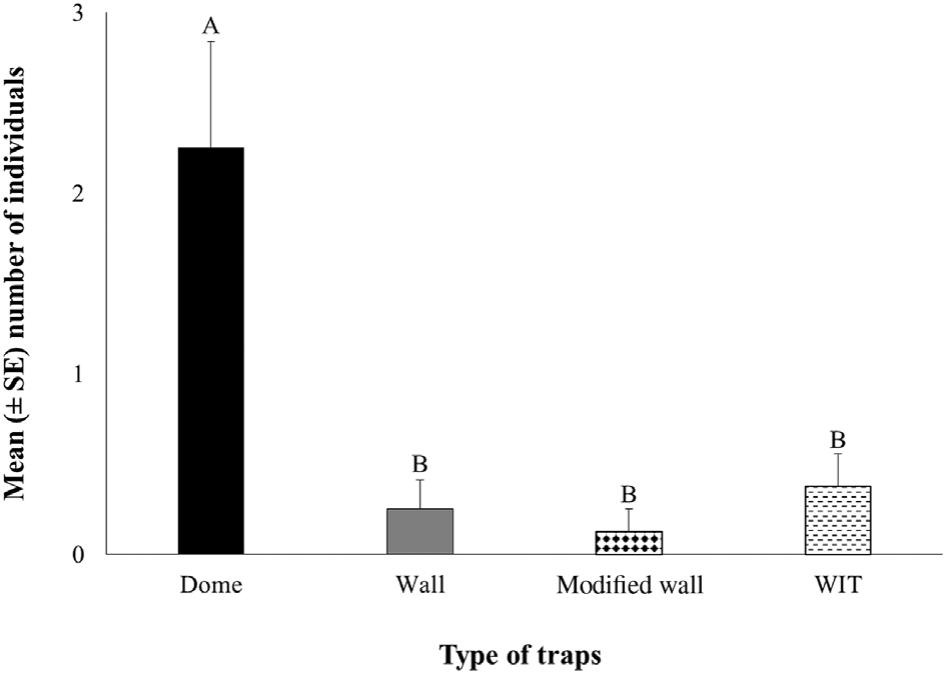
The mean (± standard error [SE]) number of all life stages collected for dome, wall, modified wall, and Wilkins interaction trap (WIT) traps. Means followed by the same letter do not differ significantly, according to the Kruskal–Wallis test, at *P* < 0.05. Statistical parameters were recorded with test statistics 14.406, *P* = 0.002. In all cases, df = 3 and total *N* = 32.

The mean number of individuals per life stage (larvae, pupae, and adult), depending on the trap type (dome, unmodified wall, modified wall, and WIT), is presented in Fig. 5. Only the trap was significant for the main effects and their interaction (trap × room) did not affect significantly the number of caught individuals in the traps (Table 2). Generally, most individuals were captured most often at the adult stage (Fig. 5). Significant differences were found in room 6, in which the dome trap had the highest number of captures compared to the unmodified wall, modified wall, and WIT traps (Fig. 5). However, there were no significant differences between rooms 5 and 6, according to the Mann–Whitney *U*‐test, since the same captures were found in both rooms (Table 2). The unmodified wall, modified wall, and WIT traps collected significantly fewer individuals compared to the dome trap (Fig. 6).

**Table 2:** Generalized linear model showing the main effects and their interaction of captures of *Trogoderma granarium* in traps for bioassay II.

Main effects and interactions	df	Wald *x* ^2^	*P*
Whole model	7	31.17	<0.001
Intercept	1	4.92	0.026
Trap	3	16.88	0.001
Room	1	0.00	1.000
Trap × room	2	1.20	0.546

### 3.3 Bioassay IΙI: trapping results

**Figure 7:**
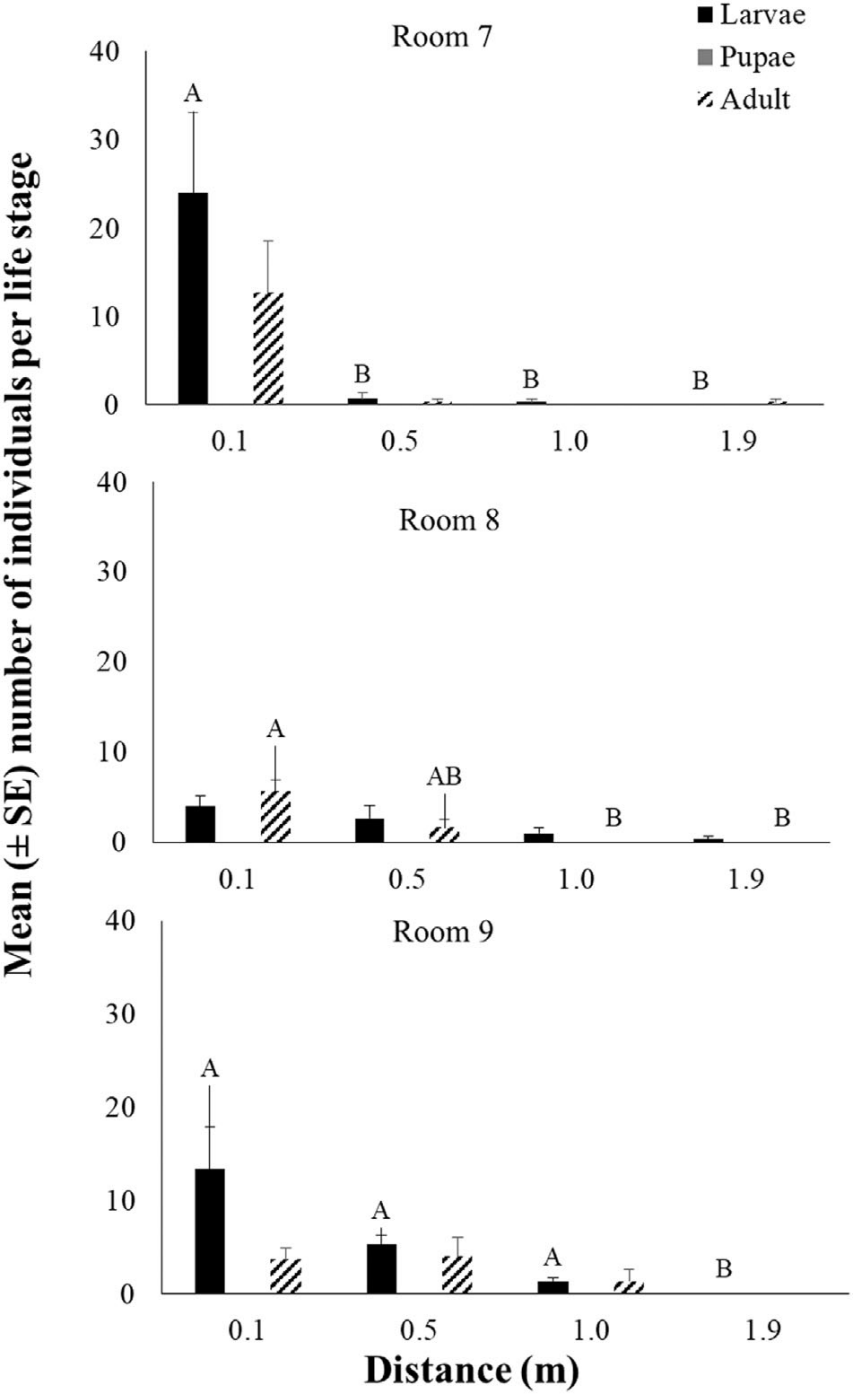
The mean (± standard error [SE]) number of larvae, pupae, and adults collected using the Wilkins interaction trap (WIT) trap for each room at different release distances. Within each room and life stage, means followed by the same letter do not differ significantly, according to the Kruskal–Wallis test, at *P* < 0.05. Where no letters exist, no significant differences were noted. Statistical parameters were recorded for room 7 at larvae (test statistic 8.194, *P* = 0.042), for room 8 at adult (test statistic 9.470, *P* = 0.024), and for room 9 at larvae (test statistic 10.385, *P* = 0.016). In all cases, df = 3 and total *N* = 12.

**Figure 8:**
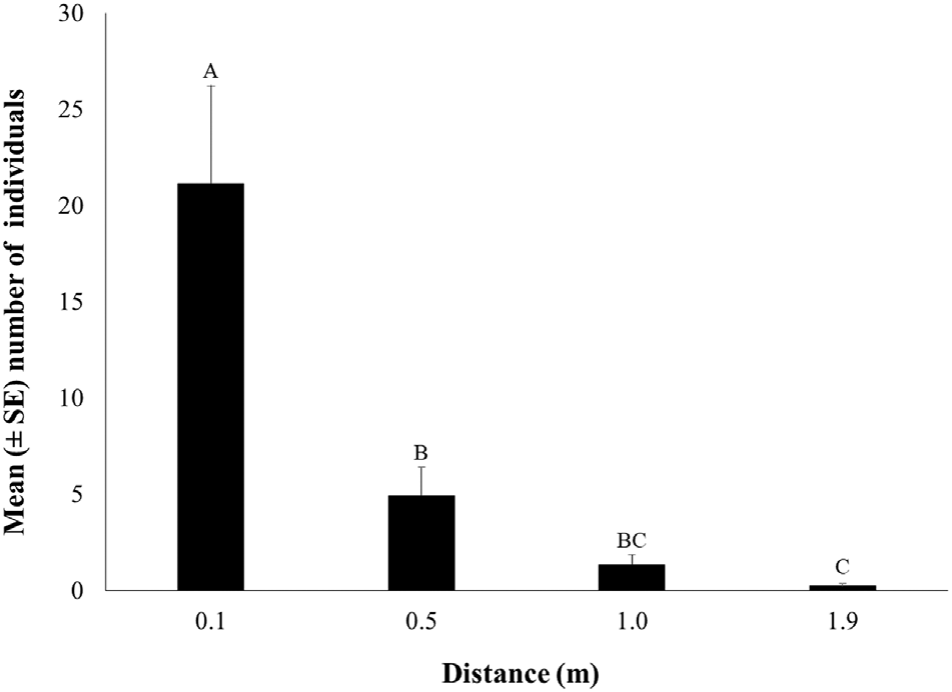
The mean (± standard error [SE]) number of all life stages collected using the Wilkins interaction trap (WIT) trap at different release distances. Means followed by the same letter do not differ significantly, according to the Kruskal–Wallis test, at *P* < 0.05. Statistical parameters were recorded with test statistics 28.823, *P* < 0.001. In all cases, df = 3 and total *N* = 36.

**Figure 9:**
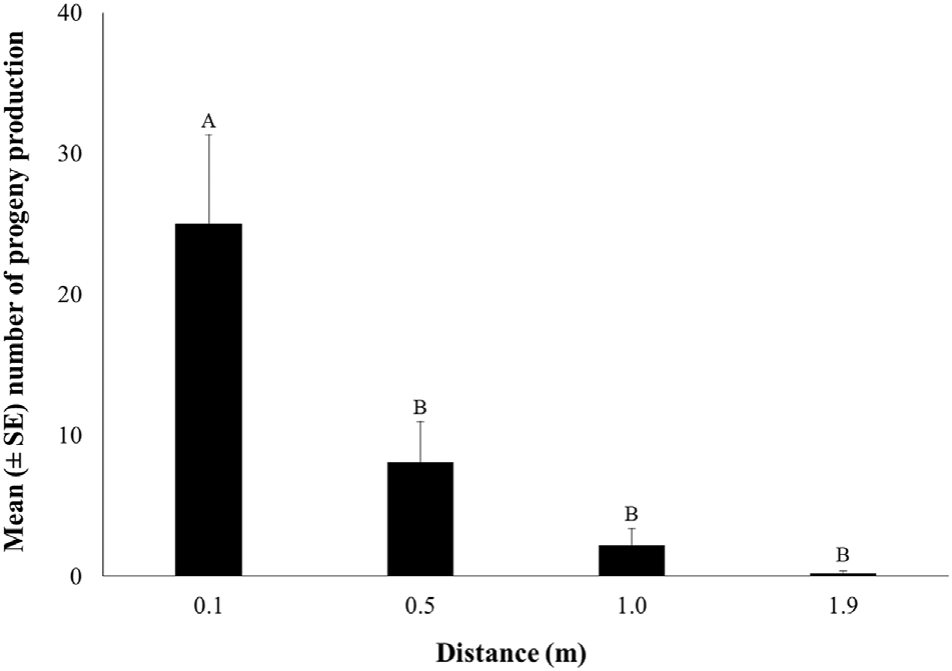
The mean (± standard error [SE]) number of progeny production (including all life stages) using the Wilkins interaction trap (WIT) trap at different release distances (0.1, 0.5, 1.0, and 1.9 m). Means followed by the same letter do not differ significantly, according to the Kruskal–Wallis test, at *P* < 0.05. Statistical parameters were recorded with test statistics 22.265, *P* < 0.001. In all cases, df = 3 and total *N* = 36.

The mean number of individuals per life stage (larvae, pupae, and adult) using the WIT trap in rooms 7, 8, and 9 at different release distances (0.1, 0.5, 1.0, and 1.9 m) is shown in Fig. 7. All main effects and their interaction (distance × room) affected significantly the number of caught of *T. granarium* in the traps (Table 3). Similarly, most individuals were collected at the larval stage (Fig. 7). The highest number of individuals was captured from the release distance of 0.1 m, while there were zero captures from the release distance of 1.9 m in all rooms (Fig. 8). More specifically, the number of larvae and adults was significantly affected by the release distance (Figs 8 and 9). Regarding progeny production, all main effects and their interaction were significant (Table 4). More than 20 individuals were found at the release distance of 0.1 m, which is significantly higher compared to the rest of the tested release distances (Fig. 9).

**Table 3:** Generalized linear model showing the main effects and their interaction of captures of *Trogoderma granarium* in traps for bioassay III.

Main effects and interaction	df	Wald *x* ^2^	*P*
Whole model	11	428.05	<0.001
Intercept	1	20.05	<0.001
Distance	3	122.63	<0.001
Room	2	13.89	0.001
Distance × room	5	29.76	<0.001

**Table 4:** Statistical parameters for the main effects and their interaction of progeny production of *Trogoderma granarium* for bioassay III.

Main effects and intercation	df	Wald *x* ^2^	*P*
Whole model	11	574.06	<0.001
Intercept	1	36.83	<0.001
Distance	3	103.93	<0.001
Room	2	21.95	<0.001
Distance × room	4	55.78	<0.001

### 3.4 Bioassay III: plume reach

**Figure 10:**
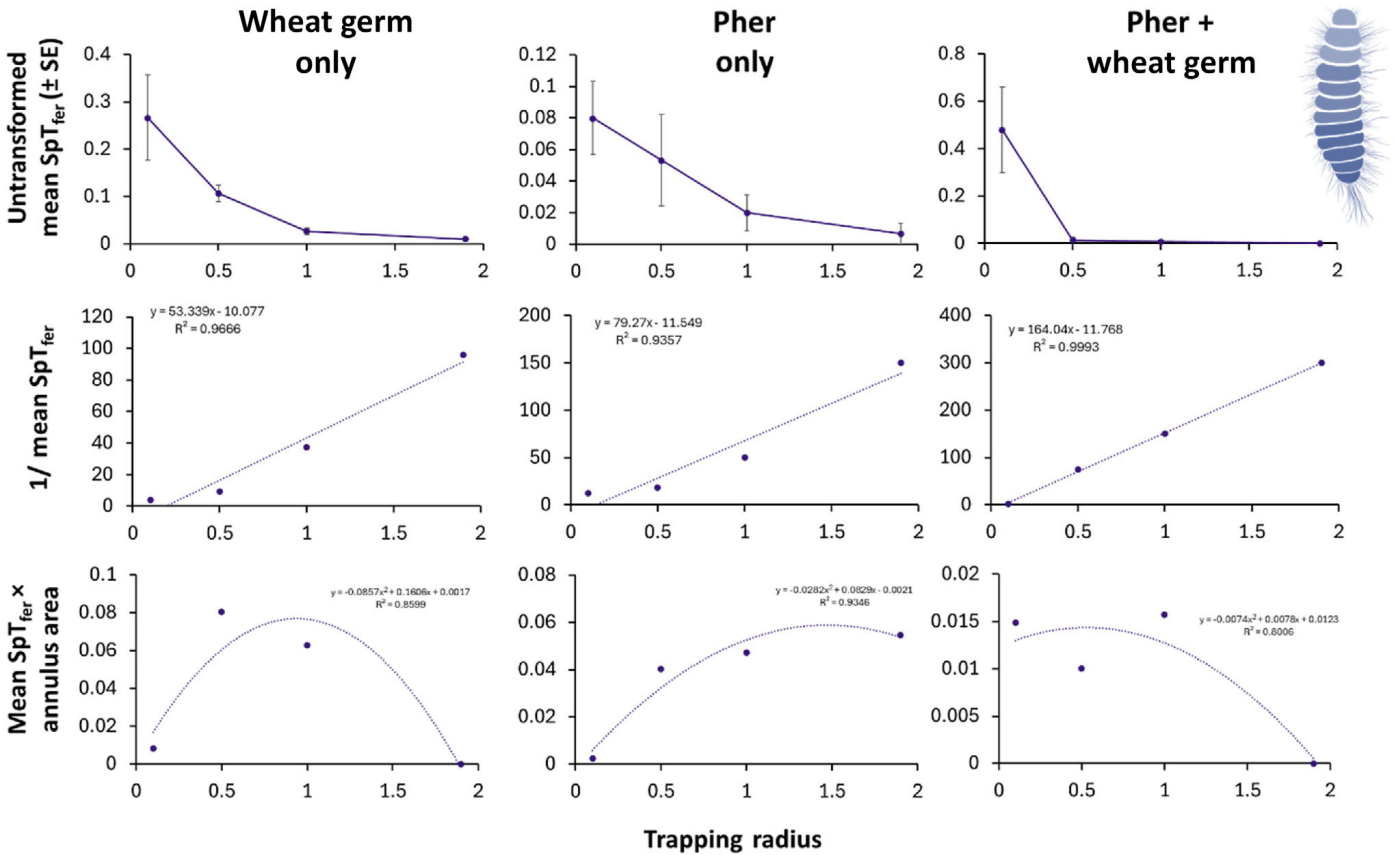
The mean proportion of larval *Trogoderma granarium* trapped (SpT_fer_, top row) in Wilkins interception traps (e.g., Wilkins interception trap [WIT]), the inverse SpT_fer_ (Miller–Adams–McGhee plots, middle row), and mean SpT_fer_ × annulus area (Miller plots, bottom row) to describe aspects of plume reach and trapping efficiency when traps were baited with wheat germ only (left column), *T. granarium* sex pheromone (middle column), or both (right column). Abbreviations: Pher ‐ sex pheromone for *Trogoderma* spp.

**Figure 11:**
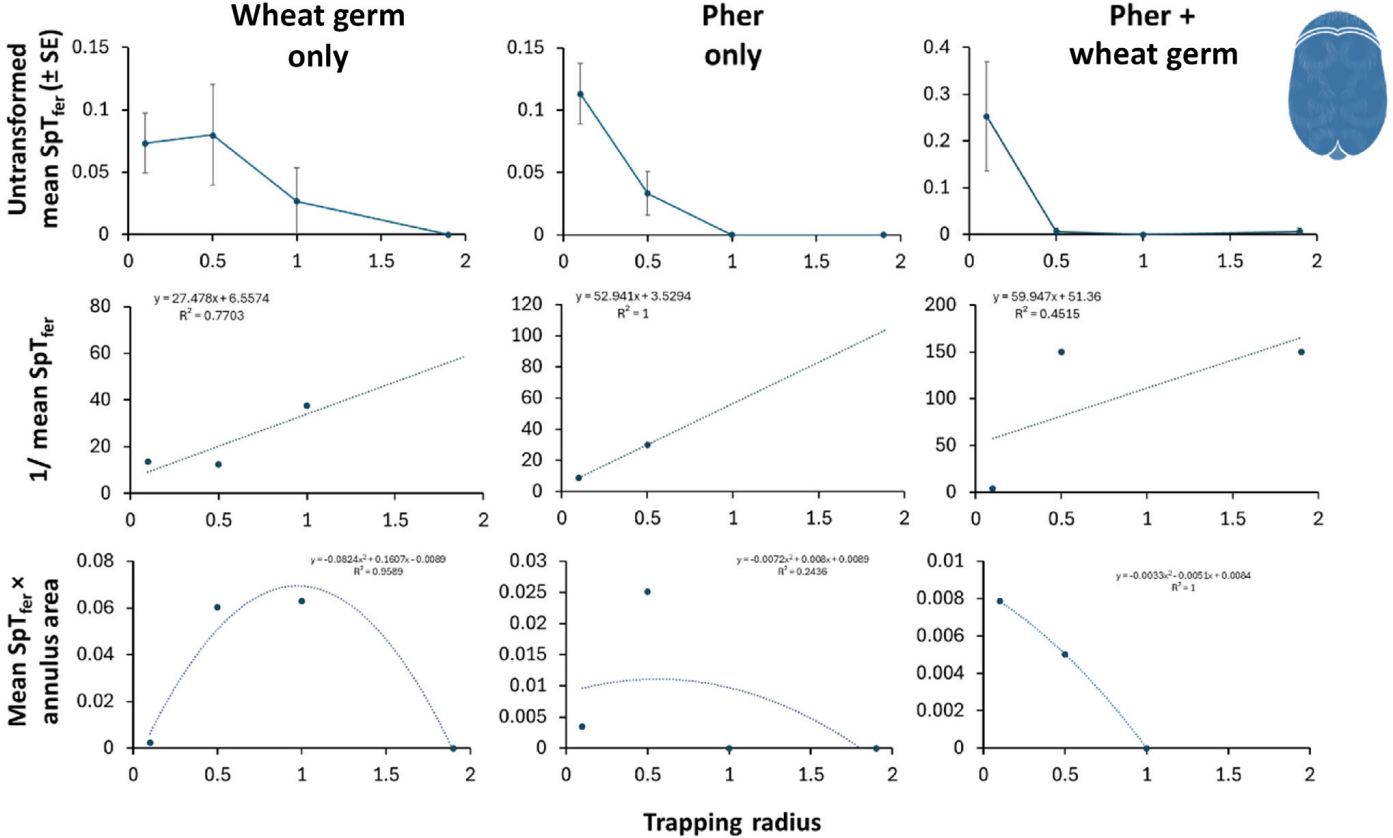
The mean proportion of adult *Trogoderma granarium* trapped (SpT_fer_, top row) in Wilkins interception traps (e.g., Wilkins interception trap [WIT]), the inverse SpT_fer_ (Miller–Adams–McGhee plots, middle row), and mean SpT_fer_ × annulus area (Miller plots, bottom row) to describe aspects of plume reach and trapping efficiency when traps were baited with wheat germ only (left column), *T. granarium* sex pheromone (middle column), or both (right column). Abbreviations: Pher ‐ sex pheromone for *Trogoderma* spp.

We found that the plume reach of the WIT trap ranged between 2.62 and 5.71 cm for adult and 0.96–2.94 cm for larval *T. granarium* depending on semiochemical treatment (Table 5, and Figs 10 and 11). The mean plume reach of the WIT trap was 3.77 ± 0.98 cm for adult and 1.96 ± 0.99 cm for larval *T. granarium*. The fitted lines on the MAG plots have negative *y*‐intercept values and the slopes are very large, which provides further support that the plume reach is small for the WIT trap in the case of *T. granarium*. The dynamics of SpT_fer_ (proportion of *T. granarium* trapped) exhibit nonlinearity. The Miller plots in Figs 10 and 11 imply that greatest or most effective captures of *T. granarium* larvae and adults are around 1 m with drop‐off before and afterwards. The trapping radius of the WIT trap to capture 95% of the released *T. granarium* was 1.88–2.96 m depending on semiochemical treatment and life stage (Table 4). The WIT trap covers a trapping area of 10.1–27.5m^2^ for *T. granarium* depending on life stage and semiochemical treatment.

**Table 5:** Summary of the plume reach and trapping radius of Wilkins interception trap (e.g., WIT) for *Trogoderma granarium* calculated using the MAG Slope as presented in Miller *et al*. 15.

Life stage	Room no	Trt	MAG^a^ slope	*R* ^2^ ^b^	*L* ^c^	Plume reach (cm)	Trapping radius^d^ (m)	Trapping area (m^2^)
Larvae	7	Pheromone + wheat germ	164	0.999	0.04	0.96	1.91	11.5
Adults	7	Pheromone + wheat germ	60	0.452	0.10	2.62	2.54	20.3
Larvae	8	Pheromone only	79	0.936	0.08	1.98	2.96	27.5
Adults	8	Pheromone only	52.9	0.999	0.12	2.97	1.79	10.1
Larvae	9	Wheat germ only	53.3	0.967	0.12	2.94	1.88	11.1
Adults	9	Wheat germ only	27.5	0.770	0.23	5.71	1.89	11.2

^a^ MAG, Miller‐Adams‐McGhee slope from MAG Plots in Miller *et al*.^41^.

^b^ *R* ^2^, regression coefficient.

^c^ Uncorrected *L* (length corresponding to trap diameter) calculated as 2π divided by the slope from MAG plot from Miller *et al*.^41^.

^d^ The trapping radius is calculated from Miller plots where the fitted function intercepts zero on the *x* axis.

## 4 DISCUSSION

The results provide the first direct comparison between floor and wall traps for *T. granarium*. It is noteworthy that none of the modified wall trap designs worked. This can be easily explained by their closer proximity to the release of the insects and the fact that vertical climbing is not needed. However, it is also revealing that in the first bioassay, in which insects were not reclaimed and removed in between trials as they were in the second bioassay, the wall traps performed relatively better. In the continuing release situation, the dome traps were approximately twice as effective as the wall traps. However, when the insects were removed each week the dome traps were nearly 10 times more effective. These data further support that insects find these traps largely by exploration of the area and are not strongly actively attracted by long‐range cues.

One of the key findings of the present study was that the distance from the trap/attractant source is an important parameter that can affect trapping efficacy. Based on our results, the response of *T. granarium* individuals is best when these individuals are in the vicinity of the trap. Such a pattern is to be expected and has been reported in the case of other stored product insect species as well.^37^, ^38^, ^39^ For *L. serricorne*, a species that responds strongly to pheromone‐baited traps, Arbogast *et al*.^37^ showed that there was competition mediated by distance for adults between the pheromone source and the food supply. In our attract‐and‐kill work, we found that most dead individuals are within the vicinity of the trap (0.1 m). We also have recorded dead *T. granarium* adults in a wider zone, which may suggest that adults are affected more than larvae. This agrees with the findings of Gourgouta *et al*.,^32^ who reported that *T. granarium* adults respond better than larvae in floor traps. Indeed, the plume reach of WIT traps was generally found to be greater for adults than for larvae in our study.

Taking into account the current data, as well as previous datasets from laboratory experiments,^32^, ^33^ there is still work to be done to optimize traps and/or attractants to improve the behavioral response of *T. granarium* individuals. In a similar semifield establishment for the evaluation of an insecticide‐coated net for the control of *L. serricorne* and the tobacco moth, *Ephestia elutella* (Hübner) (Lepidoptera: Pyralidae), Rumbos *et al*.^40^ noted a considerable response to the pheromone‐baited traps used. In our case, dome traps performed consistently better than the other traps used, including the unmodified wall trap and its modified version, which both performed similarly. This may suggest that the poor behavioral response of *T. granarium* individuals is due to the inability of traps to act as ‘arrestants’ in a given area, and the inability of the species to approach and enter the trap, as they do not prefer to climb on an inclined surface.^35^ This should be taken seriously into account in conjunction with the cryptic behavior of this species, which seems to be able to remain unnoticed even when a trapping network is deployed. This is supported by our estimation of the plume reach, which is small. Indeed, according to the nonlinear dynamics in mean SpT_fer_ (proportion of *T. granarium* trapped), *T. granarium* is sensitive to very small changes in distance from the trap at close range to the trap,^41^ but capture is insensitive over longer distances to the trap. The Miller plots (bottom rows, Figs 10 and 11) indicate that *T. granarium* is a random walker and not a ballistic or near‐ballistic mover, which is suggested by a peak in most of the curves, followed by a smooth downturn. This is significant because more random walkers than ballistic movers will intercept a trap under identical conditions; ballistic movers move in a straight line, whereas random walkers will also approach a trap laterally, from the rear, and frontally.^41^ Conversely, we found that larvae cannot escape easily when captured in the trapping device, which was evident from the large number of pupae that were found within the traps.

These considerations naturally affect the development of attract‐and‐kill designs, which underlines the need for improved ‘attract’ modifications. On the other hand, the ‘kill’ can be achieved with different active ingredients and techniques, which have proved effective for the control of *T. granarium*.^3^, ^5^, ^42^ For instance, Athanassiou *et al*.^3^ and Kavallieratos *et al*.^5^ found that this species is susceptible to a wide range of contact insecticides with different modes of action, ranging between the pyrrole chlorfenapyr and the organophosphate pirimiphos‐methyl. For some of these insecticides, the authors found that there was a considerable delayed mortality of the exposed individuals after their removal from the toxic agent (pirimiphos‐methyl and deltamethrin), which could be utilized further in attract‐and‐kill studies. However, the low trap recapture rate in these rooms, even without competing sources of odors, highlights the problem with detecting and managing populations with such a strategy. A new cue would need to be a superstimulus and unique among the cues present in a food facility or port of entry.^43^, ^44^

A new aspect of this study was estimating the plume reach and trapping radius for an existing trap with various stimuli in the literature^24^ using a widely accepted method.^41^ Estimating the plume reach and trapping efficiency has been done in the recent past with other quarantine and/or invasive species, such as *Halyomorpha halys* (Stål) (Hemiptera: Pentatomidae),^45^ to much success. In that case, despite the fact that *H. halys* flies 2–2.5 km in a 24‐h period with long‐distance dispersers flying 117 km,^46^ the plume reach for highly effective traps and pheromonal stimuli was less than 3 m. Our study shows the utility of this method and that it can be emulated to evaluate the active space of traps and attractants for other species of stored product insects. This will deliver improved knowledge on the chemical ecology of essential tools that food facility stakeholders rely on, and they can then be systematically improved.

Our data provide some evidence for traps and attractants that can be used for the detection of *T. granarium*, along with some follow‐up issues to address that require further improvement. In light of the current findings, detection of *T. granarium* individuals in real‐world conditions is still problematic because of the poor to moderate response of adults and larvae of this species to the trapping devices used here. The simultaneous presence of other species of this genus in the same area that is to be monitored for the occurrence of *T. granarium* is expected to complicate further the overall monitoring effort, despite the fact that there are no negative results in trapping from the co‐occurrence of more than one *Trogoderma* spp. The evaluation of additional improvements is necessary both in terms of trap design and the attractants within the trap.

## Data Availability

The data that support the findings of this study are available from the corresponding author upon reasonable request.
